# OpenEvidence

**DOI:** 10.29173/jchla29960

**Published:** 2026-08-03

**Authors:** Julia Martyniuk, Cara McNeil

**Affiliations:** Liaison and Education Librarian, University of Toronto, Toronto, ON, Canada; Graduate Student Library Assistant, University of Toronto, Toronto, ON, Canada

**Product:** OpenEvidence

**URL:** https://www.openevidence.com/

## Purpose

OpenEvidence is a web-based artificial intelligence (AI) clinical decision making tool for clinicians and health care providers. It is described as being an “AI copilot for doctors that helps them make high-stakes decisions at the point of care” [1]. The software is a search aid designed to provide clinicians and health care workers with a point-of-care answer to natural language search prompts (including clinical questions as well as patient case details). OpenEvidence aims to “redefine” evidence-based medicine, claiming the tool’s ability to quickly incorporate and synthesize new and emerging research would enable clinicians “the power to make faster, more evidence-based decisions” [2].

In a July 2025 press release, OpenEvidence states that clinicians using the tool receive “point-of-care answers grounded in the latest research, complete with references” [2]. The chief executive officer (CEO) of OpenEvidence, Daniel Nadler, has described the product not as an “answer engine” but as a search engine, with sources always provided for every query [3].

## Product description

**Fig. 1:**
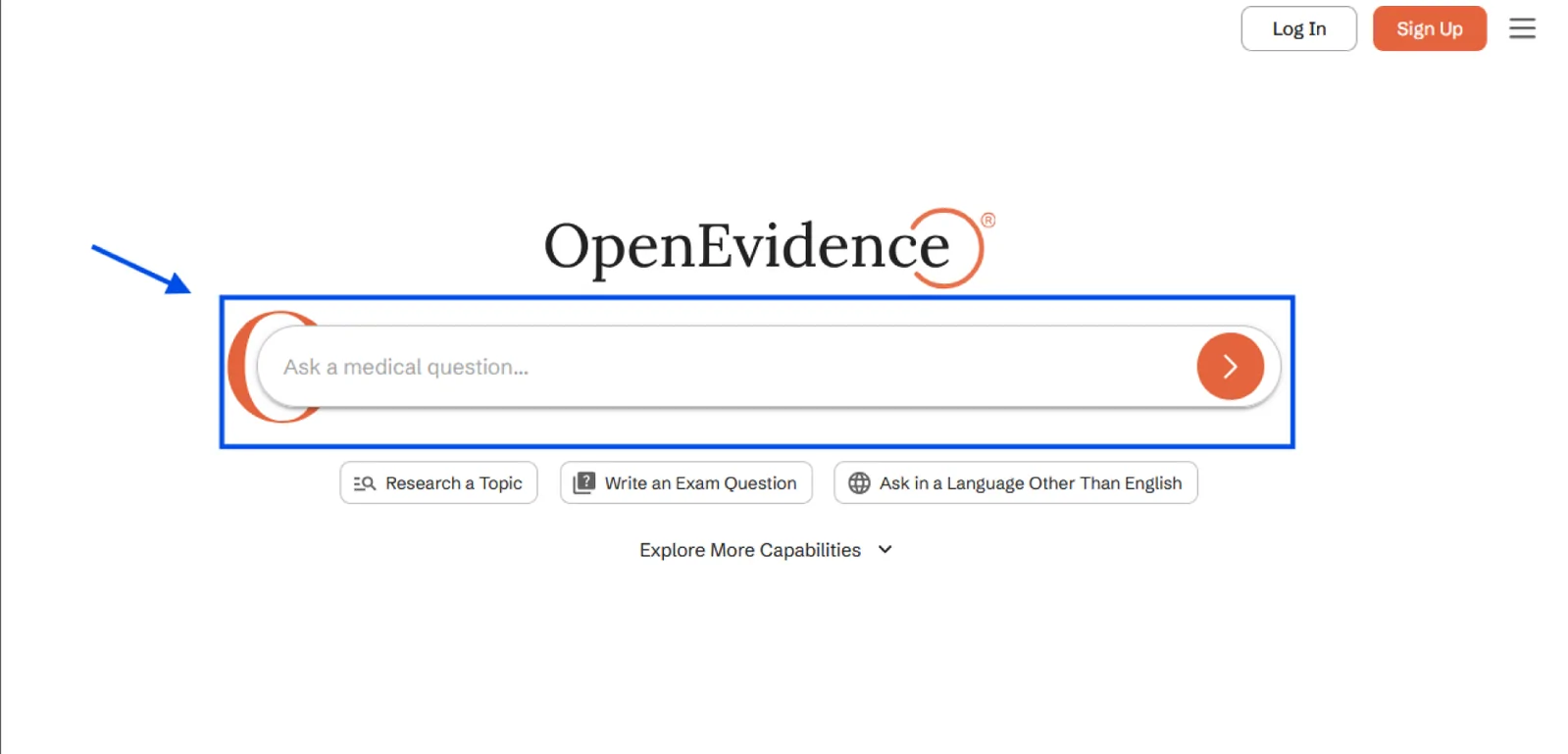
OpenEvidence browser search view for an unverified user

In both the registered account version and non-registered account (public) version, to start using OpenEvidence a user must type a query into the search bar (Figure 1).

OpenEvidence allows users to format their query using natural language (similar to a Google search), in contrast to most databases that require keyword or concept searching. Due to its ability to process natural language queries, OpenEvidence can handle long multifaced queries within a single search, including patient case details (which may include multiple conditions) and numerous different interventions. Once the query is processed (which can take some time), a summary of the research and a linked reference list is provided as a result. At the bottom of the results, users can select an auto-generated follow up question or ask their own follow up question in a second search bar.

## Intended audience

OpenEvidence is free for verified health care professionals and health care students in Canada and the United States and requires proof of occupational status either through physicians’ Medical Identification Number, a copy of board certification documents, or a verification printout from a recognized certifying body. In order to complete registration, it is required to select “I attest that I am a licensed healthcare professional and all information is true and accurate” [4]. Nadler has stated OpenEvidence is only intended to be used by physicians, not patients or the general public [3].

## Special features

**Fig. 2:**
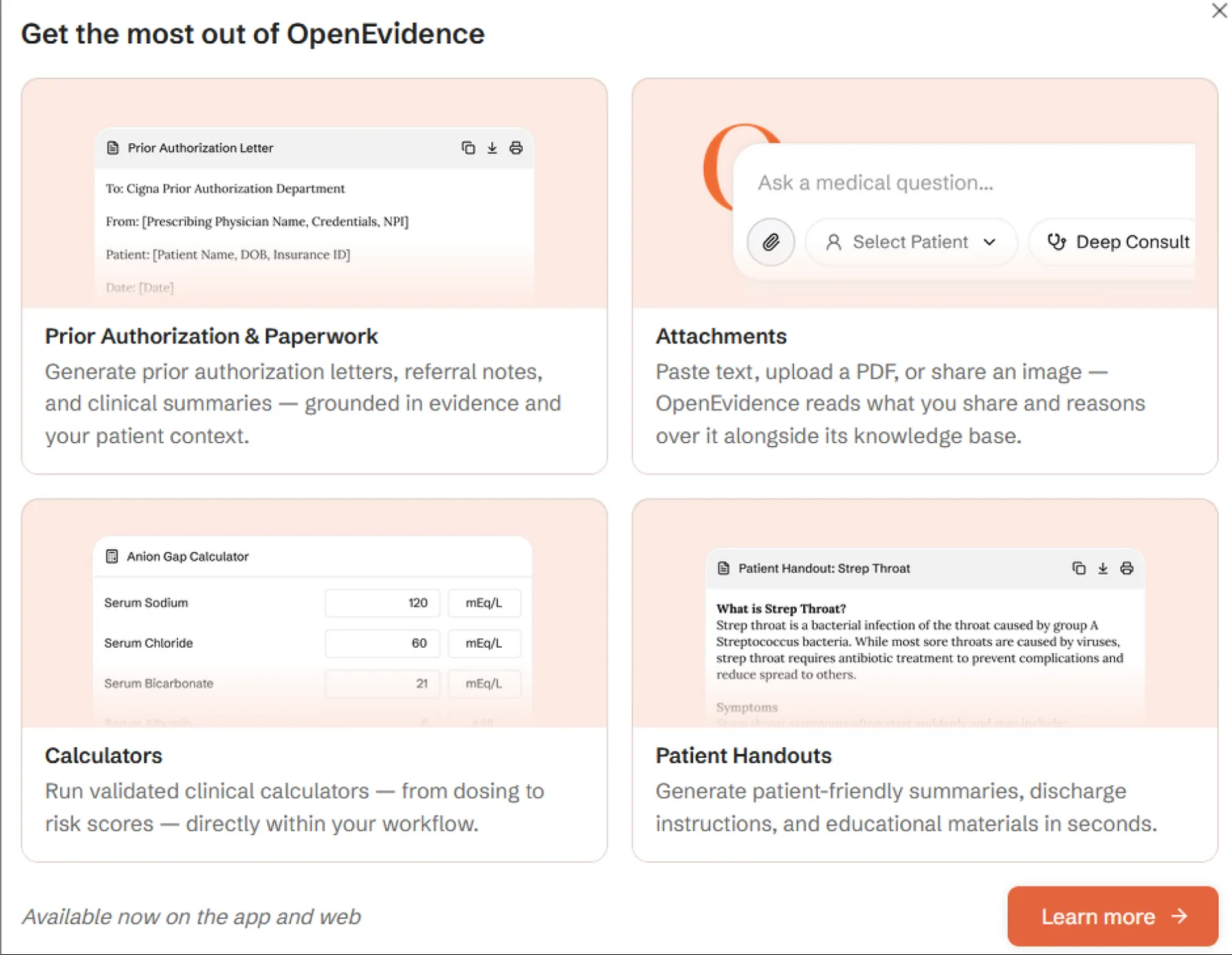
Tutorial pop-up for additional OpenEvidence features: Prior Authorization & Paperwork, Attachments, Calculators, Patient Handouts

In addition to medical searches, OpenEvidence also offers registered users tools related to administrative and clinical workflow use cases, including adding attachments, drafting patient handouts, diagnostic reasoning support, support for over 50 clinical calculators, drug monograph modules, and guideline-based modules (Figure 2) [5]. OpenEvidence also claims to support all input languages.

The OpenEvidence main page provides a list of sample prompts for users to explore its capabilities, including inquiring about drug dosing, guidelines, writing exam questions, and constructing workups.

**Fig. 3:**
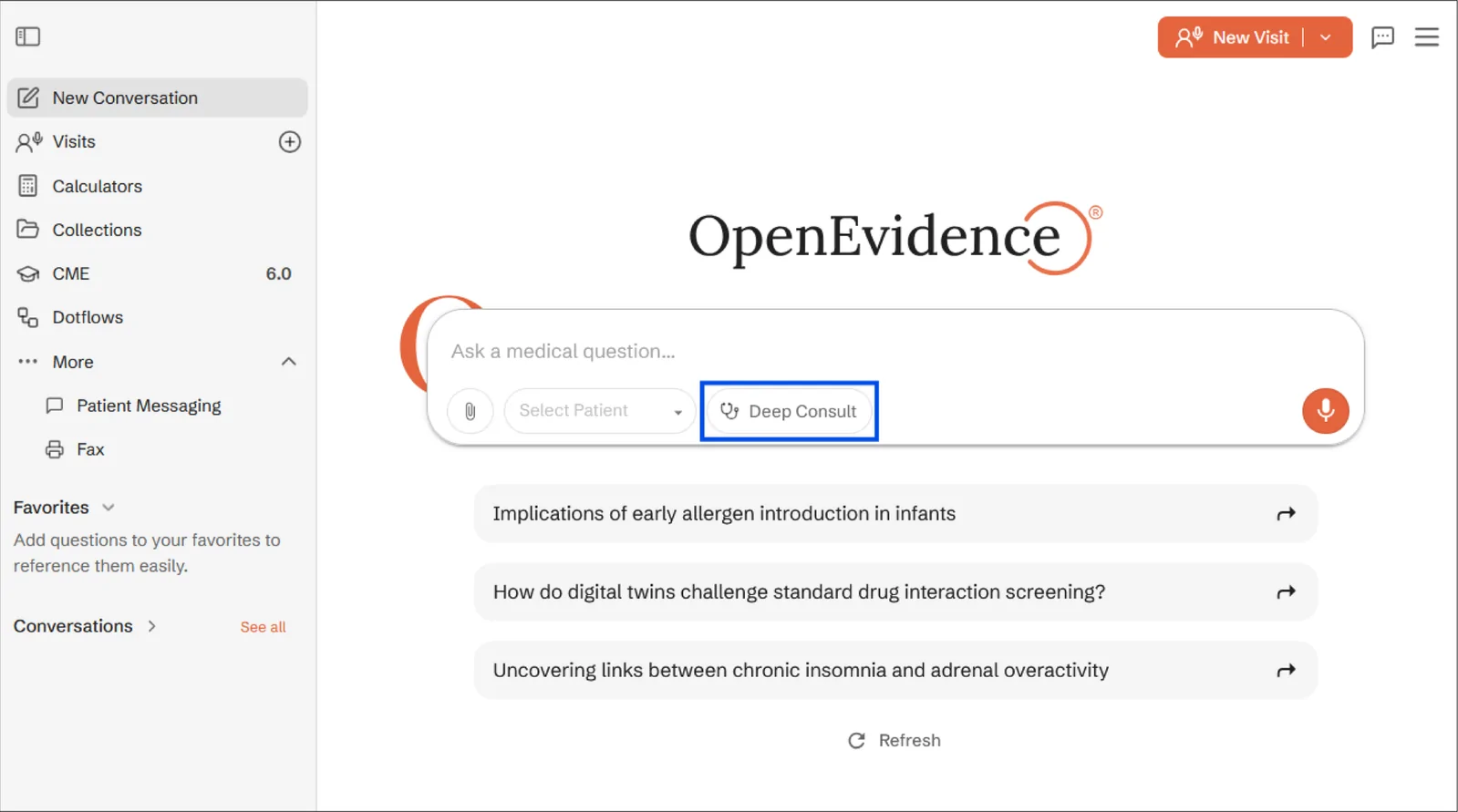
OpenEvidence browser search view for a verified user. Includes access to Deep Consult and other tools listed on the left.

In mid-2025 OpenEvidence released an extension of their main search called “Deep Consult”, which allows registered users to generate an in-depth report of a particular topic, claiming to mimic research that someone at a PhD level would be able to produce (Figure 3) [6].

## Platform

OpenEvidence is available for all users on a web browser, or for registered users through their proprietary app (available through the Apple app store and the Google Play store) [7].

## Usability

OpenEvidence’s search function is very straightforward and intuitive to use. If a non-medical query is asked in the search bar, (for example, “write me a poem about medicine”) the user will receive a pop-up stating, “The question is outside the scope of OpenEvidence”.

## Strengths and weaknesses

### Strengths

The algorithm that powers OpenEvidence sets itself apart by claiming to be a “specialized clinical model” [8], which in this case is a Large Language Model (LLM) which focuses on peer-reviewed medical science as its basis for training [9].

Nadler claims that OpenEvidence has only been trained on sources the company has free access to under copyright law. This originally involved any resources freely accessible from the American government (such as medical science from the Food and Drug Administration (FDA) and the Centers for Disease Control and Prevention (CDC)), and eventually expanded to licencing deals with third parties such as the New England Journal of Medicine (NEJM) [9].

If assertions about training data are true, this would make OpenEvidence one of the only major generative AI tools on the market not at risk of copyright violations (unlike ChatGPT). It would also imply that generated responses should be more consistently medically relevant than other models which are trained on non-medical data (such as the open internet).

### Weaknesses

While OpenEvidence is Health Insurance Portability and Accountability Act (HIPAA) compliant [10], it makes no claims to be compliant with the Canadian equivalent legislation Personal Information Protection and Electronic Documents Act (PIPEDA). Their Terms of Use states “[i]f you access our Services from outside the United States, you are responsible for compliance with applicable local laws”, thus the onus is on the user to ensure PIPEDA requirements are being met [11].

The American disposition of OpenEvidence also extends to search results–default answers often assume the user to be a practitioner from the United States. One must specify within the search that they are not American to receive results more relevant to a Canadian context.

OpenEvidence, like many other AI search engines, suffers from a lack of reproducibility and transparency. The same question asked on different computers, different days, or different accounts can yield different results each time, which is a deficiency when compared to traditional point-of-care tools. When the same question was run on the same account one month apart, OpenEvidence’s “deep consult” response included 10 new references, with a slightly wider date range (Table 1).

**Table 1:** Comparison of OpenEvidence’s and UpToDate’s responses to the query “cervical screening guidelines for female patients between the age of 18-30". Two OpenEvidence “DeepConsult” responses were compared, one generated on 15 April 2026 and one generated on 13 May 2026.

Feature	OpenEvidence	OpenEvidence	UpToDate
Author List & Disclosures	No	‘’	Yes
Cost	Free	‘’	$579/year
Follow Up Questions	Yes	‘’	No
Graphics	Yes	‘’	Yes
Currency	Date of generation (15 April 2026)	Date of generation (13 May 2026)	Topic last updated (26 March 2026)
References Provided	13	23	74
References Date Range	2018-2026	2017-2026	1984-2026
References Geography	92% from American sources (12/13)	83% from American sources (19/23)	68% from American sources (50/74)
References Recency	54% from the past 2 years (7/13)	52% from the past 2 years (12/23)	22% from the past two years (16/74)

## Comparison with similar products

With over 3 million healthcare providers using it worldwide, UpToDate is a leading competitor in the evidence-based medicine software industry [12]. Using an inquiry for “cervical screening guidelines for female patients between the age of 18-30”, we compared OpenEvidence’s “deep consult” response with the top recommended article found using UpToDate (Table 1).

## Currency

As of April 2026, OpenEvidence has signed licencing deals with the NEJM, the Journal of the American Medical Association (JAMA) Network, The National Comprehensive Cancer Network, Wiley, and Cochrane [13–16]. It was confirmed by the CEO that the language model used for OpenEvidence has been trained on the full text of NEJM [9], however it’s unclear from press releases if this is also the case for all other licencing deals.

Since the release of OpenEvidence 2.0 in December 2024, the platform regularly integrates new administrative and clinical support tools for doctors to use.

## Cost

OpenEvidence is available to verified health care professionals and health care students in Canada with an account for unlimited, free access due to the platform’s use of advertising [17]. Individuals without an account (unverified users) are able to use a limited version of OpenEvidence, only through the web browser, and are restricted to a weekly query limit.
